# Decreased Mortality Rate Among COVID-19 Patients Prescribed Statins: Data From Electronic Health Records in the US

**DOI:** 10.3389/fmed.2021.639804

**Published:** 2021-02-03

**Authors:** Ivana Marić, Tomiko Oskotsky, Idit Kosti, Brian Le, Ronald J. Wong, Gary M. Shaw, Marina Sirota, David K. Stevenson

**Affiliations:** ^1^Department of Pediatrics, Stanford University School of Medicine, Stanford, CA, United States; ^2^Department of Pediatrics, UCSF, San Francisco, CA, United States; ^3^Bakar Computational Health Sciences Institute, UCSF, San Francisco, CA, United States

**Keywords:** SARS-CoV-2, COVID-19, statins, electronic health records, inflammatory response, oxidative stress, mortality rate

## Abstract

The severe respiratory illness due to SARS-CoV-2, the virus responsible for coronavirus disease 2019 (COVID-19), is triggered by an intense pro-inflammatory host response. Statins, prescribed primarily for lipid reduction, are known to have anti-inflammatory and immunomodulatory properties and have been associated with a reduced mortality rate among COVID-19 patients taking statins as reported in two recent retrospective studies. However, a meta-analysis that included nine studies showed that statin use did not improve in-hospital outcomes of those with COVID-19. In addition, concerns regarding the use of statins and an increase in COVID-19 infections have been raised, as statins may increase the expression of angiotensin-converting enzyme 2 (ACE2), the primary receptor for the SARS-CoV-2 virus. Our goal was to investigate the effect of statins in COVID-19 patients in a large, diverse patient population across the United States containing nearly 120,000 patients diagnosed with COVID-19. We used propensity score matching of demographics, comorbidities, and medication indication to compare statin-treated patients (*N* = 2,297) with matched controls (*N* = 4,594). We observed a small, but statistically significant, decrease in mortality among patients prescribed statins (16.1%) when compared with matched COVID-19-positive controls (18.0 to 20.6%). These results support previous evidence that statins do not increase COVID-19-related mortality and may, in fact, have a mitigating effect on severity of the disease reflected in a slight reduction in mortality. Mixed findings on effects of statins in COVID-19 patients reported in the literature should prompt prospective randomized controlled trials in order to define better who might be advantaged with respect to clinical outcomes.

## Introduction

In less than a year since its outbreak, the coronavirus disease 2019 (COVID-19) pandemic has taken over one million lives to date (1). The severity of COVID-19 symptoms in patients is strongly associated with being older and having pre-existing medical conditions (2). The severe respiratory illness of COVID-19 is primarily triggered by an intense pro-inflammatory host response (3). Statins—medications routinely prescribed for cholesterol and lipid lowering—are also known to have anti-inflammatory and immunomodulatory properties, capable of reducing inflammatory responses and oxidative stress (4–6). Furthermore, several observational studies have indicated that statin use may be effective in reducing mortality and hospitalization due to viral infections, such as influenza (7–9). Statins have also been shown to be beneficial for patients with various autoimmune inflammatory conditions (5) *via* several pathways.

The potential capability of statins to reduce the severity of COVID-10 outcomes has been investigated recently in several retrospective studies. Specifically, in a study of 13,981 COVID-19 patients in Hubei Province, China (10) and in a smaller study of 154 elderly patients in nursing home residents in Belgium (11), the use of statins was associated with a reduced mortality rate. However, a meta-analysis that included nine studies (for a total of 3,449 patients) showed that statin use did not improve in-hospital outcomes of those with COVID-19 (12). Furthermore, an increased mortality rate was observed among COVID-19 patients with Type-2 diabetes taking statins (13). In addition, concerns regarding the use of statins and an increase in COVID-19 infections have been raised, as statins can increase the expression of angiotensin-converting enzyme 2 (ACE2), the primary receptor for the SARS-CoV-2 virus (14). Electronic health records (EHR) provide an opportunity to study therapeutic effects on a population level. Our goal was to leverage data from electronic health records covering a comprehensive population of nearly 120,000 COVID-19 patients to investigate the potential effects of statin use in COVID-19 patients in a large, diverse population in the United States.

## Methods

**Figure 1:**
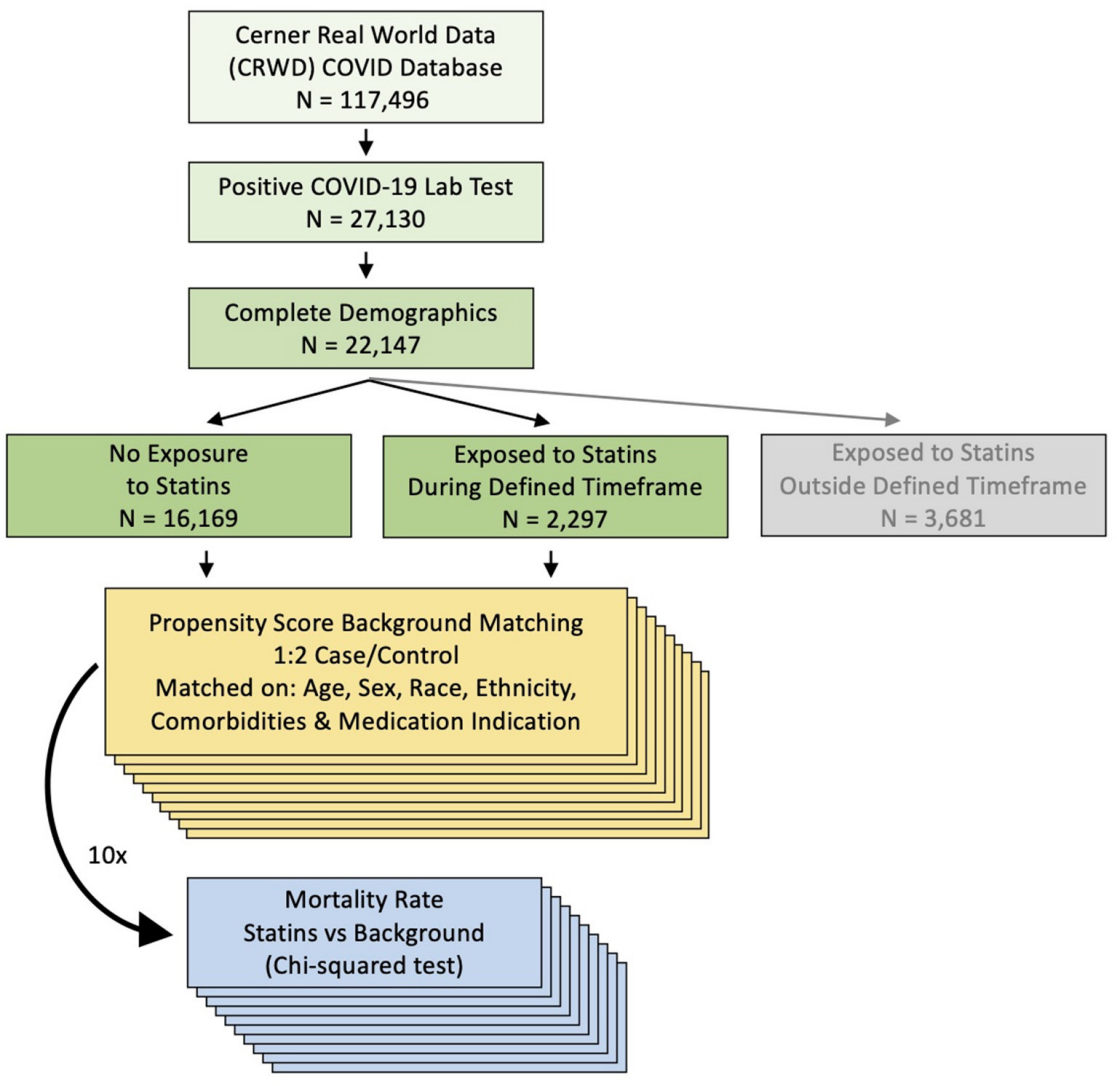
Flow chart of the patient inclusion and the method.

Data were obtained and analyzed from Cerner's large COVID-19 EHR database, which contains records of 117,496 COVID-19 patients across 62 healthcare centers as of July 2020. Deidentified Cerner Real-World Data is extracted from the EHR of hospitals in which Cerner has a data use agreement. Encounters include admissions, clinical and microbiology laboratory, pharmacy (medication orders and dispensing), and billing information, which are all date and time stamped, providing a temporal relationship between treatment patterns and clinical information. All patients had an emergency room visit or were hospitalized. Only patients with a COVID-19 diagnosis confirmed by a laboratory test for SARS-CoV-2 (including nucleic acid amplification tests and immunoassays) and with known values for demographics (age at encounter, sex, race and ethnicity) between February 2020 and July 2020 were included in analyses. Among 27,130 patients with a positive lab test, 22,147 had complete demographic information (Figure 1). COVID-19 patients with a medication order for a statin with an order status “active” or “completed” and without a designation of “as needed” (i.e., medication taken only when needed) at least once within a period of 10 days before and seven days after testing positive for COVID-19, were compared with COVID-19 patients who have no medication orders for statins. Individuals who were exposed to statins outside of this period of 10 days before and 7 days after testing positive for COVID-19 were excluded from our analysis (*N* = 3,681), resulting in total of *N* = 18,466 patients in the final cohort (Figure 1). If there was more than one positive laboratory test for COVID-19 for an individual, then the date of the first positive test was used. The following statins were included: atorvastatin (Lipitor), cerivastatin (Baycol), fluvastatin (Lescol), lovastatin (Mevacor), pitavastatin (Zypitamag, Livalo or Nikita), pravastatin (Pravachol), rosuvastatin (Ezallor or Crestor), simvastatin (FloLipid or Zocor). Considered comorbidities, identified by using *International Classification of Diseases, 9*^*th*^ *and 10th Revision, Clinical Modification* (ICD-9/10) diagnosis codes, included hypertension (I10, I11, I12, I13, I15, I16, 401-405), diabetes (O24, E11, E10, E13, 250), COPD (J44, 491.2, 493.2, 496), obesity/high BMI (E66, 278, Z68.25, Z68.26, Z68.27, Z68.28, Z68.29, Z68.3X, Z68.4, V85.2, V85.3, V85.4), high cholesterol/hyperlipidemia (E78.0, E78.1, E78.2, E78.3, E78.4, E78.5, 272, 277), and atherosclerotic cardiovascular disease (I20, I21, I22, I23, I24, I25, 410-414, I63, I64, I65, I66, I69.3, 437.0, 437.1, G45.9, 433-435, 435.9). Body Mass Index (BMI) values were identified by Logical Observation Identifiers Names and Codes (LOINC) code 39156-5.

### Statistical Analysis

Propensity score matching (PSM) (15) with a nearest neighbor method and a 1:2 ratio was used to match statin-treated patients with controls. The R Matchit package was used for the analysis (16). Three PSMs were performed. The first matching was based on demographics (age, sex, ethnicity, race). The second matching was based on demographics and on comorbidities [hypertension, diabetes, obesity/high BMI, chronic obstructive pulmonary disease (COPD)]. The third matching included—in addition to all variables from the first two matchings—indications for a statin prescription (e.g., high cholesterol, hyperlipidemia, atherosclerotic cardiovascular disease). Welch two-sample two-sided *t*-test was performed to evaluate if there was a significant difference in the mean age when comparing the statin-treated group and the untreated group. Two-sample two-sided Mann–Whitney *U* test with continuity correction was performed to evaluate if there was a significant difference in BMI when comparing the statin-treated and untreated group. Pearson's Chi-squared test with Yates' continuity correction was performed to examine the association between statin use and the outcome of death in patients with COVID-19. For each of the three matchings, we carried out 10 iterations and evaluated mortality rate as follows. Each iteration included all statin patients and a subset of controls chosen by PSM. Due to a large number of controls, several controls could have had a same value of the propensity score value and therefore, some statin patients could be matched to more than two controls. To explore this variability and show the robustness of the results, 10 iterations were performed by varying the control patients who had a tie in their propensity scores.

## Results

A total of 18,466 patients with a laboratory-confirmed COVID-19 diagnosis and complete demographic characteristic were identified, after filtering out those who were prescribed statins outside of the defined timeframe (Figure 1). We found that 2,297 patients had an order for statins during the defined timeframe, and 16,169 patients had no orders for statins. Cohort characteristics are shown in Table 1. After matching, the mean age among statin-treated patients (68.4 years) was similar to that of controls for the three matchings (matching 1: 68.4 years, *p* = 0.82; matching 2: 68.6, *p* = 0.54; matching 3: 68.4, *p* = 0.93). The median BMI among statin-treated patients (29.04 kg/m^2^) was higher than the median BMI of controls for the three matchings (matching 1: 27.85–28.00 kg/m^2^, *p* < 1E-08; matching 2: 28.30–28.40 kg/m^2^, *p* < 1E-02; matching 3: 28.18–28.24 kg/m^2^, *p* < 1E-02); however, the calculated difference in BMI between the statin-treated and untreated groups was small (matching 1: 1.04–1.19 kg/m^2^; matching 2: 0.64–0.74 kg/m^2^; matching 3: 0.80–0.86 kg/m^2^) (Supplementary Table 1). Among statin-treated patients, the mortality rate was 16.1%. For the 10 iterations of PSM by demographics, we observed a higher mortality rate among controls in each iteration (18.0 to 18.4%, Chi-squared test, *p* < 0.05 for 9 out of 10 iterations). Exact mortality rates and *p*-values for each iteration are shown in Table 2. When carrying out 10 iterations of PSM by comorbidities as well as demographics, we also observed a significantly higher mortality rate in the controls (20.0 to 20.3%) in each iteration (Chi-squared test, *p* < 1.00E-03 for all iterations) (Table 3). When carrying out 10 iterations of PSM by medication indication as well as demographics and comorbidities, we again observed a significantly higher mortality rate in the controls (20.3 to 20.6%) in each iteration (Chi-squared test, *p* < 1.00E-04 for all iterations) (Table 4).

**Table 1:** Cohort characteristics.

	**On Statin**		**Overall**	
**Characteristic**	***N* (total = 2,297)**	**%**	***N* (total = 18,466)**	**%**
**Age**				
18–34	17	0.7%	4,581	24.8%
35–49	166	7.2%	4,104	22.2%
50–64	684	29.8%	4,800	26.0%
65+	1,430	62.3%	4,981	27.0%
**Sex (%)**				
Male	1,222	53.2%	8,848	47.9%
Female	1,075	46.8%	9,618	52.1%
**Race**				
American Indian or Alaska Native	40	1.7%	532	2.9%
Asian or Pacific islander	91	4.0%	594	3.2%
Black or African American	742	32.3%	5,378	29.1%
Mixed racial group	232	10.1%	2,985	16.2%
Other racial group				
White	1,192	51.9%	8,977	48.6%
**Ethnicity**				
Hispanic or Latino	630	27.4%	6,555	35.5%
Not Hispanic or Latino	1,667	72.6%	11,911	64.5%
**Condition**				
Hypertension	1,833	79.8%	7,481	40.5%
Diabetes	1,254	54.6%	4,365	23.6%
COPD	321	14.0%	1,171	6.3%
Obesity/High BMI	694	30.2%	4,163	22.5%
High cholesterol/Hyperlipidemia	1,530	66.6%	3,849	20.8%
ASCVD	898	39.1%	2,406	13.0%
**Outcome**				
Death	369	16.1%	1,619	8.8%

**Table 2:** Propensity score matching by demographics: a mortality rate, associated X-squared and *p*-value in each iteration.

**Cohort**	**Permutation**	**N deceased**	**N total**	**Mortality rate**	**Chi-squared**	***P*-value**
on Statin	1	369	2,297	16.06%	4.05	**0.0443**
control	1	829	4,594	18.05%		
on Statin	2	369	2,297	16.06%	5.36	**0.0206**
control	2	843	4,594	18.35%		
on Statin	3	369	2,297	16.06%	4.31	**0.0378**
control	3	832	4,594	18.11%		
on Statin	4	369	2,297	16.06%	5.56	**0.0183**
control	4	845	4,594	18.39%		
on Statin	5	369	2,297	16.06%	4.50	**0.0340**
control	5	834	4,594	18.15%		
on Statin	6	369	2,297	16.06%	4.13	**0.0420**
control	6	830	4,594	18.07%		
on Statin	7	369	2,297	16.06%	4.31	**0.0378**
control	7	832	4,594	18.11%		
on Statin	8	369	2,297	16.06%	4.22	**0.0399**
control	8	831	4,594	18.09%		
on Statin	9	369	2,297	16.06%	3.79	0.0516
control	9	826	4,594	17.98%		
on Statin	10	369	2,297	16.06%	5.56	**0.0183**
control	10	845	4,594	18.39%		

*Bold indicates p < 0.05*.

**Table 3:** Propensity score matching by demographics and comorbidities: a mortality rate, associated X-squared and *p*-value in each iteration.

**Cohort**	**Permutation**	**N deceased**	**N total**	**Mortality rate**	**Chi-squared**	***P*-value**
on Statin	1	369	2,297	16.06%	16.71	**4.37E-05**
control	1	927	4,594	20.18%		
on Statin	2	369	2,297	16.06%	17.38	**3.05E-05**
control	2	931	4,594	20.27%		
on Statin	3	369	2,297	16.06%	15.06	**1.04E-04**
control	3	917	4,594	19.96%		
on Statin	4	369	2,297	16.06%	16.20	**5.69E-05**
control	4	924	4,594	20.11%		
on Statin	5	369	2,297	16.06%	15.71	**7.39E-05**
control	5	921	4,594	20.05%		
on Statin	6	369	2,297	16.06%	15.71	**7.39E-05**
control	6	921	4,594	20.05%		
on Statin	7	369	2,297	16.06%	17.38	**3.05E-05**
control	7	931	4,594	20.27%		
on Statin	8	369	2,297	16.06%	15.87	**6.77E-05**
control	8	922	4,594	20.07%		
on Statin	9	369	2,297	16.06%	16.54	**4.77E-05**
control	9	926	4,594	20.16%		
on Statin	10	369	2,297	16.06%	17.21	**3.34E-05**
control	10	930	4,594	20.24%		

*Bold indicates p < 0.05*.

**Table 4:** Propensity score matching by demographics, comorbidities and indication for a statin prescription: a mortality rate, associated X-squared and *p*-value in each iteration.

**Cohort**	**Permutation**	**N deceased**	**N total**	**Mortality rate**	**Chi-squared**	***P*-value**
on Statin	1	369	2,297	16.06%	18.60	**1.61E-05**
control	1	938	4,594	20.42%		
on Statin	2	369	2,297	16.06%	18.96	**1.34E-05**
control	2	940	4,594	20.46%		
on Statin	3	369	2,297	16.06%	18.08	**2.12E-05**
control	3	935	4,594	20.35%		
on Statin	4	369	2,297	16.06%	17.56	**2.79E-05**
control	4	932	4,594	20.29%		
on Statin	5	369	2,297	16.06%	18.43	**1.77E-05**
control	5	937	4,594	20.40%		
on Statin	6	369	2,297	16.06%	17.73	**2.55E-05**
control	6	933	4,594	20.31%		
on Statin	7	369	2,297	16.06%	20.40	**6.27E-06**
control	7	948	4,594	20.64%		
on Statin	8	369	2,297	16.06%	18.78	**1.47E-05**
control	8	939	4,594	20.44%		
on Statin	9	369	2,297	16.06%	19.49	**1.01E-05**
control	9	943	4,594	20.53%		
on Statin	10	369	2,297	16.06%	17.73	**2.55E-05**
control	10	933	4,594	20.31%		

*Bold indicates p < 0.05*.

## Discussion

We observed a small, but statistically significant, decrease in mortality among COVID-19 patients prescribed statins when compared with propensity-score matched controls. Importantly, we did not find an increase in mortality associated with statin use. Our findings are in agreement with previous results indicating that statins may reduce COVID-19-related mortality (10, 11). The mean age of statin-treated patients in the current study (68.4 years) was lower than that in the Belgium study (85.6 years) and comparable to that in the Wuhan study (66.0 years). Ours is the first study performed using a large, diverse population across the United States. In contrast, an increased mortality rate was observed among COVID-19 patients with type-2 diabetes taking statins (13). One possible explanation is that this cohort focused only on high-risk patients hospitalized for COVID-19 who had Type-2 diabetes and therefore were at a higher risk for complications. Although ACE2 expression has been reported as variable in Type-2 diabetic patients independent of statin use (17), it is conceivable that high ACE2 expression in some Type-2 diabetics might have been further increased with statin use, increasing the potential for viral entry into cells (17–19).

Several mechanisms by which statins may benefit COVID-19 patients have been proposed, although not proven. A recent study suggested that statins could be efficient inhibitors of the SARS-CoV-2 main protease (Mpro), a key coronavirus enzyme (19). Inhibition of the MYD88–NF-κB pro-inflammatory pathway, blockage of the NLRP3 inflammasome, and upregulation of the stress-response protein, heme oxygenase (HO-1) (4, 11, 20–25) have also been suggested. After entering the cell through ACE2, SARS-CoV-2 can cause a downregulation of ACE2 (20) and a pro-inflammatory host response *via* the TLR-MYD88–NF-κB pathway, causing increased cytokine levels, inhibition of HO-1, and coagulation dysfunction (20, 26, 27). Statins have been shown to modulate the MYD88–NF-κB proinflammatory pathway, upregulate ACE2, and have anti-thrombotic properties, mainly through their effects on platelet function, all potentially important effects on patients with severe cases of COVID-19 (20, 23).

In addition, several recent studies have proposed the direct modulation of HO-1 as a potential therapeutic intervention for COVID-19 (28–30) as it can potentially prevent a “cytokine storm,” a dysfunctional anti-inflammatory response (26). Statins can upregulate HO-1 leading to the production of iron (Fe^2+^), carbon monoxide (CO) and biliverdin, which is rapidly reduced to bilirubin (31–34). These bioactive products have immunomodulatory, antioxidative, anti-inflammatory, vasodilatory, and anti-apoptotic properties, as well as anti-thrombotic properties, such as the inhibition of platelet aggregation and adhesion. Further support for the hypothesis that there might be therapeutic benefits of statins for COVID-19 patients is based on a known role of HO-1 in inducing type-1 interferon (IFN) expression and an established role of IFNs in inhibiting replication of various viruses including coronaviruses (28).

Finally, the gene expression of HO-1 is affected by the number of glutathione thymidine (GT) dinucleotide repeats in the HO-1 promoter region (26), which greatly varies among populations. The presence of longer (GT)n repeats are associated with lower basal HO-1 expression, and a decrease response to a noxious stimulus, such as infection or any other myriad of factors that can trigger upregulation of the gene (35). Because these individuals cannot sufficiently up regulate HO-1, to regain a non-inflamed state after an inflammatory stimulus (a normal physiologic response), possibly contributing to exacerbated acute or more chronic autoimmune inflammatory syndromes. The importance of there being differences in capacity to upregulate HO-1 among people is that it is plausible that those individuals with long (GT)n repeats could be at greater risk for a “cytokine storm.” Thus, statins might be useful in boosting the expression of HO-1 in these individuals when they present with COVID-19. Interestingly, a baby born totally deficient in HO-1 died in infancy with multi-system organ failure caused by a systemic vasculitis (36). While statins vary in their intended effectiveness of lipid reduction, as a class of drugs they share the above-described mechanistic generic qualities.

One of the main limitations of our report is that it is, like others, retrospective, allowing us to only demonstrate an association between statin use and COVID-19 mortality, but not causal effects. Moreover, while records are available from 2015 and beyond for some individuals in this database, this was not the case for all individuals and as such, pertinent information including previous medication use and comorbidities for some individuals could be incomplete in this database. Statins can lower plasma LDL; however, we were unable to determine if there was a significant difference in baseline LDL levels between our statins-treated and untreated groups due to the low percentage (19 to 30%) of each group that has LDL values in the Cerner database (data not shown). Our study involved medication orders for statins with order statuses that ensured that the medications were administered to the individuals—an advantage compared to outpatient prescriptions where there can be uncertainty as to whether an individual fills the prescription or takes the medication. However, the administration of different statins and at various doses, which could have differing pleiotropic effects in lieu of their designed cholesterol-lowering effect, were not considered. Nonetheless, that any mitigating effect on COVID-19-related mortality was found is intriguing. While we considered several demographics and comorbidities known to be associated with COVID-19 outcomes, unaccounted confounding variables could alter this observed association.

Overall, our findings are reassuring in that the use of statins was not associated with an increased mortality among elderly patients with COVID-19. However, the observation that there might be some increased risk associated with statin use in individuals with Type-2 diabetes warrants some caution. Considering what has been speculated about the potential beneficial effects of statins and what retrospective findings have been reported, our results should motivate further prospective studies to elucidate the potential mechanisms by which statins might be protective in some COVID-19 patients or harmful in others. Statins are widely used, low-cost medications that, if proven an effective mitigating treatment, could be an affordable option to reduce the mortality of COVID-19 even in low-income countries.

## Data Availability Statement

The data analyzed in this study is subject to the following licenses/restrictions: This was an observational study of Electronic Health Records that cannot be made publicly available. Requests to access these datasets should be directed to the Cerner Clinical Research Team, coviddatalab@cerner.com.

## Author Contributions

DS proposed the hypothesis investigated in the paper and the main idea of the study, supervised the analysis, co-wrote the first draft of the manuscript with IM. MS acquired data and supervised the analysis. IM designed and performed the initial analysis for the study, co-wrote the first draft of the manuscript with DS. TO acquired data, performed the analysis, made the figure and tables, contributed to writing the manuscript. GS supervised the analysis. RW contributed to writing the manuscript. IK and BL contributed to the analysis. All authors discussed results, provided critical feedback and contributed to the final manuscript.

## Conflict of Interest

MS is a scientific advisor at twoXAR. The remaining authors declare that the research was conducted in the absence of any commercial or financial relationships that could be construed as a potential conflict of interest.
