# Supplementary material for: Decreased Mortality Rate Among COVID-19 Patients Prescribed Statins: Data From Electronic Health Records in the US
Source: Front Med (Lausanne). 2021 Feb 3;8:639804. doi: 10.3389/fmed.2021.639804 (PMC7887302; doi:10.3389/fmed.2021.639804)
Supplement: Supplementary file 1 [file Table_1.pdf]

	On Statin BMI						Control BMI							
Permu-tation	N case with BMI value (N total = 2297)	**mean (kg/m2)**	std dev (kg/m2)	min (kg/m2)	**median (kg/m2)**	max (kg/m2)	N control with BMI value (N total = 4594)	**mean (kg/m2)**	std dev (kg/m2)	min (kg/m2)	**median (kg/m2)**	max (kg/m2)	(Case median) - (Control median) (kg/m2)	Mann Whitney U, p-value
**Matching 1**														
1	2034	**31.72**	16.97	14.102	**29.04**	273	3855	**30.14**	14.68	9.5	**27.9**	260	1.14	6E-10
2	2034	**31.72**	16.97	14.102	**29.04**	273	3789	**30.15**	14.71	9.5	**27.93**	260	1.11	2E-09
3	2034	**31.72**	16.97	14.102	**29.04**	273	3848	**30.27**	15.97	9.5	**27.96**	260	1.08	2E-10
4	2034	**31.72**	16.97	14.102	**29.04**	273	3867	**30.31**	15.70	9.5	**27.92**	260	1.12	1E-09
5	2034	**31.72**	16.97	14.102	**29.04**	273	3884	**30.39**	15.85	9.5	**27.945**	256	1.095	5E-09
6	2034	**31.72**	16.97	14.102	**29.04**	273	3854	**30.16**	14.87	9.5	**27.93**	256	1.11	8E-10
7	2034	**31.72**	16.97	14.102	**29.04**	273	3856	**30.23**	14.80	9.5	**28**	260	1.04	5E-09
8	2034	**31.72**	16.97	14.102	**29.04**	273	3858	**30.24**	15.35	9.5	**27.85**	268.21	1.19	7E-10
9	2034	**31.72**	16.97	14.102	**29.04**	273	3862	**30.17**	14.76	9.5	**27.955**	260	1.085	4E-09
10	2034	**31.72**	16.97	14.102	**29.04**	273	3864	**30.20**	14.79	9.5	**28**	260	1.04	4E-09
**Matching 2**														
1	2034	**31.72**	16.97	14.102	**29.04**	273	4014	**31.41**	17.30	9.5	**28.345**	260	0.695	4E-03
2	2034	**31.72**	16.97	14.102	**29.04**	273	4029	**31.42**	17.70	9.5	**28.3**	260	0.74	2E-03
3	2034	**31.72**	16.97	14.102	**29.04**	273	4022	**31.45**	17.35	9.5	**28.4**	260	0.64	7E-03
4	2034	**31.72**	16.97	14.102	**29.04**	273	4040	**31.46**	17.42	9.5	**28.4**	260	0.64	6E-03
5	2034	**31.72**	16.97	14.102	**29.04**	273	4023	**31.45**	17.57	9.5	**28.34**	260	0.7	4E-03
6	2034	**31.72**	16.97	14.102	**29.04**	273	4030	**31.46**	17.57	9.5	**28.39**	260	0.65	4E-03
7	2034	**31.72**	16.97	14.102	**29.04**	273	4025	**31.44**	17.47	9.5	**28.33**	260	0.71	4E-03
8	2034	**31.72**	16.97	14.102	**29.04**	273	4017	**31.43**	17.22	9.5	**28.36**	260	0.68	7E-03
9	2034	**31.72**	16.97	14.102	**29.04**	273	4005	**31.47**	17.38	9.5	**28.4**	260	0.64	6E-03
10	2034	**31.72**	16.97	14.102	**29.04**	273	4024	**31.36**	17.07	9.5	**28.35**	260	0.69	3E-03
**Matching 3**														
1	2034	**31.72**	16.97	14.102	**29.04**	273	4008	**30.96**	16.34	11.95	**28.23**	256	0.81	8E-05
2	2034	**31.72**	16.97	14.102	**29.04**	273	4021	**30.95**	16.16	11.95	**28.2**	256	0.84	1E-04
3	2034	**31.72**	16.97	14.102	**29.04**	273	4000	**30.98**	16.34	11.95	**28.22**	256	0.82	1E-04
4	2034	**31.72**	16.97	14.102	**29.04**	273	4008	**30.93**	15.80	11.95	**28.245**	239.77	0.795	2E-04
5	2034	**31.72**	16.97	14.102	**29.04**	273	4000	**30.96**	16.34	11.95	**28.175**	256	0.865	8E-05
6	2034	**31.72**	16.97	14.102	**29.04**	273	4005	**30.96**	16.34	11.95	**28.18**	256	0.86	7E-05
7	2034	**31.72**	16.97	14.102	**29.04**	273	4024	**30.99**	16.31	11.95	**28.2**	256	0.84	2E-04
8	2034	**31.72**	16.97	14.102	**29.04**	273	4005	**30.90**	15.93	11.95	**28.2**	239.77	0.84	7E-05
9	2034	**31.72**	16.97	14.102	**29.04**	273	4015	**30.88**	15.75	11.95	**28.19**	239.77	0.85	1E-04
10	2034	**31.72**	16.97	14.102	**29.04**	273	4016	**30.93**	15.93	11.95	**28.22**	239.77	0.82	1E-04
